# New semi-analytical solution of the problem of vapor bubble growth in superheated liquid

**DOI:** 10.1038/s41598-020-73596-x

**Published:** 2020-10-05

**Authors:** A. A. Chernov, A. A. Pil’nik, I. V. Vladyko, S. I. Lezhnin

**Affiliations:** 1 Novosibirsk State University, Novosibirsk, Russia 630090; 2 Kutateladze Institute of Thermophysics, Siberian Branch of the Russian Academy of Sciences, Novosibirsk, Russia 630090

**Keywords:** Energy science and technology, Physics

## Abstract

This paper presents a mathematical model of the vapor bubble growth in an initially uniformly superheated liquid. This model takes into account simultaneously the dynamic and thermal effects and includes the well-known classical equations: the Rayleigh equation and the heat conductivity equation, written with consideration of specifics associated with the process of liquid evaporation. We have obtained a semi-analytical solution to the problem, which consists in reducing the initial boundary value problem with a moving boundary to a system of ordinary differential equations of the first order, valid in a wide range of operating parameters of the process at all its stages: from inertial to thermal, including the transitional one. It is shown that at large times this solution is consistent with the known solutions of other authors obtained in the framework of the energy thermal model, in particular, for the high Jacob numbers, it is consistent with the Plesset–Zwick solution.

## Introduction

The growth of a vapor bubble is one of the main processes determining boiling, and it is a complex problem described by the equations of hydrodynamics and heat transfer. Obviously, the detailed study of the mechanism of this process is of great importance for both theory and practice. The history of numerous attempts to obtain the law of vapor bubble growth dates back more than a century, beginning with classical works^1–6^ and ending with modern research^7–11^. They were based either on highly simplified mathematical models^12,13^, or on a fairly complete system of classical equations as applied to the problem under consideration, solved numerically^14–18^. Similar studies can be found for gas bubbles^19–24^ with the only difference that the mass transfer processes are the governing ones instead of the heat transfer processes.

In the most general statement, the growth of a vapor bubble is determined by various factors: dynamic, kinetic, thermal, etc. Depending on the properties of the two-phase system under consideration, at some stage of the process, the factors that determine it change. Therefore, to solve the problem analytically, various assumptions are used, which allow the construction of simplified mathematical models with one main mechanism of the process control. Let us list the the most common models^25^.

*Dynamic inertial model* In this model, the vapor pressure in the bubble during the entire process is kept constant and equal to the saturation pressure at the initial liquid temperature, and the pressure drop determines the bubble growth. The temperature of liquid at the bubble boundary does not change and equals the temperature at a great distance from the bubble, i.e., the effect of liquid cooling near the interface due to evaporation is neglected. The analytical solution under these assumptions is obtained by simple integration of the Rayleigh equation. In this model, the dependence of the bubble radius on time is linear.

*Dynamic viscous model* In this model, the bubble growth rate is limited by the viscous forces. Obviously, such a model can be applied only to highly viscous liquid media. The initial equation for this limiting case is obtained from the Rayleigh equation (neglecting the inertial terms), which is easily integrated. The dependence of the bubble radius on time in this model is exponential.

*Energy molecular-kinetic model* In this model, an attempt to take into account the kinetics of phase transformation is made. The bubble growth rate in this case is determined, among other things, by the rate of liquid evaporation from the superheated interface. At that, the vapor in the bubble during its growth is in the unsaturated state.

*Energy thermal model* This model is most common in the literature. In this model, the bubble growth is determined by the supply of heat to the interface from external superheated layers of liquid. At that, all supplied heat is spent on evaporation. This requires knowledge of temperature distribution in the liquid volume. The vapor pressure in the bubble throughout the process is kept constant and equal to the pressure of the surrounding liquid. The vapor in the bubble is in the saturated state. The dependence of the bubble radius on time in this model has the root nature. Moreover, the proportionality coefficient (which is sometimes called the growth modulus) is a function of the Jacob number and the vapor – liquid density ratio and it is found by different authors in different ways. The most famous is the Plesset–Zwick formula^1^, which is valid for high Jacob numbers. A sufficiently complete analytical solution to the problem in integral form was obtained by Scriven^4^. Along with this, various empirical approximation dependencies are widely used^26,27^.

It is obvious that the energy thermal model, despite its widespread use, as well as all other mathematical models, has a limited scope. In particular, a number of approximations made in this model lead to a solution with an infinite rate of the bubble growth at the initial time, and this is physically incorrect. In this regard, the attempts to create various hybrid models that take into account simultaneously the dynamic and thermal effects are being made. For this, the Rayleigh equation should be solved together with the heat conductivity equation, which, as practice shows, is associated with significant difficulties. In this paper, we tried to find a relatively simple semi-analytical solution that would simultaneously describe both inertial and thermal effects and which would become an alternative to direct numerical calculations.

## Problem statement

### Main equations

Let us consider the growth of a single supercritical vapor bubble, formed at the initial time moment in uniformly superheated liquid. The system of equations describing the behavior of such a bubble includes well-known classical equations written in relation to the problem under consideration, taking into account the specifics associated with the process of liquid evaporation. When stating the problem, we will use the following assumptions. The temperature and vapor pressure in the bubble are uniform. The vapor in the bubble is stationary and it stays saturated throughout the process. The liquid around the bubble is viscous and incompressible. The flow is spherically symmetric.

The velocity field around the bubble is obtained from the continuity equation and has the following form:

1 $$\begin{aligned} v_l(r) = v_{l R} (R/r)^2, \end{aligned}$$

where $v_l$ is the radial velocity of liquid, *r* is the radial coordinate with the beginning in the center of the bubble and *R* is bubble radius. Hereinafter, the subscripts “*l*” and “*v*” refer to the liquid and vapor phases, respectively and subscript “*R*” refers to the value at the interface.

The equation of liquid motion can be written as

$$\begin{aligned} \frac{\partial v_l}{\partial t} + \frac{\partial }{\partial r} \ \left( \dfrac{p_l}{\rho _l} + \dfrac{v_l^2}{2}\right) = 0, \end{aligned}$$

where *t* is the time, *p* is the pressure and $\rho$ is the density. The last equation can be integrated along the radial coordinate, taking into account (1):

2 $$\begin{aligned} \dfrac{1}{R} \frac{\hbox {d}}{\hbox {d}t} \left(v_{l R} R^2\right) - \dfrac{v_{l R}^2}{2} = \dfrac{p_{l R} - p_{l i}}{\rho _l}. \end{aligned}$$

Hereinafter, superscripts “*i*” and “*f*” correspond to the initial and final states, respectively. Note, that the liquid velocity at the interface $v_{l R}$ differs from the rate of bubble growth ${\dot{R}}$ due to the phase transition: $v_{l R} = {\dot{R}} - j/\rho _l$, where *j* is the density of the mass flux at the interface and ${\dot{\square }} \equiv {\hbox {d}(\square )}/{\hbox {d}t}$ is the time derivative of arbitrary function $\square$. In the case of no phase transition, Eq. (2) becomes well-known Rayleigh equation.

The boundary conditions representing the mass, momentum and energy conservation laws can be written as follows^28^:

3 $$\begin{aligned}&\dfrac{1}{4\pi R^2} \frac{\hbox {d}}{\hbox {d}t} \ \left( \dfrac{4\pi }{3} R^3 \rho _v\right) = j; \end{aligned}$$

4 $$\begin{aligned}&p_{l R} = p_v + j v_{l R} - \dfrac{2\sigma }{R} - 4 \eta _l \dfrac{v_{l R}}{R}; \end{aligned}$$

5 $$\begin{aligned}&j {\mathscr {L}} = \lambda _l \ ({\partial T_l}/{\partial r})_{r=R}, \end{aligned}$$

where $\sigma$ is the surface tension, $\eta$ is the dynamic viscosity, $\lambda$ is the thermal conductivity, *T* is the temperature and ${\mathscr {L}}$ is the specific heat of phase transition.

The presented system of equations is closed by a thermal boundary-value problem solved inside the liquid surrounding bubble. The temperature field dynamics is described by the heat conductivity equation (volumetric heat release caused by viscous dissipation is neglected):

6 $$\begin{aligned} \frac{\partial (\rho _l {c}_l T_l)}{\partial t} + v_l \frac{ \partial (\rho _l {c}_l T_l)}{\partial r} = \dfrac{1}{r^2}\frac{\partial }{\partial r} \left( \lambda _l \, r^2 \frac{\partial T_l}{\partial r}\right) , \end{aligned}$$

where *c* is the heat capacity. Thermophysical properties of liquid will be considered constant.

At the initial moment of time, the temperature of liquid is assumed to be uniform and higher than the vapor saturation temperature at pressure $p_{l i}$ (the liquid is superheated): $(T_l)_{t=0} = T_{l i} > T^s(p_{l i})$. At a great distance from the bubble, the temperature field remains unperturbed during its growth: $(T_l)_{r\rightarrow \infty } = T_{l i}$. On the interface, the condition of local thermodynamic equilibrium is satisfied: $(T_l)_{r=R} = T^s(p_v)$ (the superscript “*s*” refers to the value of the function on the saturation line). It should be noted that the temperature of liquid at the interface changes over time.

The problem should be supplemented by the equation of vapor state in the bubble and temperature dependence of the saturation pressure in the following general form:

7 $$\begin{aligned} \rho _v&= \rho _v(p_v, T_v); \end{aligned}$$

8 $$\begin{aligned} p_v&= p^s(T_v) \quad {\text {or}} \quad T_v = T^s(p_v). \end{aligned}$$

Model equations and more accurate empirical dependencies can be used, which are numerous in the literature. We should note that dependencies (7), (8) uniquely determine functions $T^s(\rho _v)$ and $p^s(\rho _v)$, which will be used below.

According to the literature on the subject of vapor bubble growth the vapor can be considered an ideal gas and the Mendeleev–Clapeyron equation can be used as the equation of state. Moreover, to find the temperature dependence of the saturated vapor pressure, the Clapeyron–Clausius equation can be applied. It must be said that this somewhat narrows the generality of both the statement of the problem itself and the solutions obtained. However, in most cases this is enough to ensure the acceptable accuracy of the desired solution. An example of using these equations in a problem is given below.

In the framework of assumptions made, the formulated system of equations is closed and fully describes the dynamics of a vapor bubble in uniformly superheated liquid.

### Nondimensionalization

Further, we will use the following dimensionless variables: $\Theta = \dfrac{T - T_{l i}}{\Delta T_i}$ is the dimensionless temperature (varies in the process from 0 to $-1$); $\Pi = \dfrac{p - p_{l i}}{\Delta p_i}$ is the dimensionless pressure (varies in the process from 1 to 0), where $\Delta T_i = T_{l i} - T^s(p_{l i})$ is the initial superheating of liquid and $\Delta p_i = p^s(T_{l i}) - p_{l i}$ is the initial overpressure caused by superheating; $\chi = r/R$ is the dimensionless radial coordinate; ${\bar{R}} = R/R_0$; ${\bar{v}} = v/v_0$; $\tau = t/t_0$ are dimensionless radius, speed and time, respectively; ${\bar{\rho }}_v = \rho _v/\rho _l$ is the dimensionless vapor density in the bubble.

We define the characteristic size $R_0$, the speed $v_0$, and the process time $t_0$ in such a way that the characteristic time of the dynamic stage and the characteristic time of reaching the thermal stage of the bubble growth are equal: $R_0 / v_0 = R_0^2/a_l \equiv t_0$, and the Euler number $\textit{Eu}= \dfrac{\Delta p_i}{\rho _l v_0^2}$ equals 1. Here $a = \lambda /(\rho c)$ is the thermal diffusivity. In this case, $R_0 = a_l \sqrt{ \rho _l/\Delta p_i }$; $v_0 = \sqrt{\Delta p_i/\rho _l}$; $t_0 = a_l \rho _l/\Delta p_i$.

The dimensionless variables introduced in this way allow us to abstract as much as possible from the properties of liquid under consideration and to focus on the behavior of the studied dynamic system. As for the choice of characteristic values, they are most convenient for the analysis of the transition stage of the process. All similarity criteria that arise in dimensionless equations and determine the process under consideration are introduced below.

## Problem solving and analysis of results

### Semi-analytical solution

Let us turn from the variables *t* and *r* to the variables $\tau$ and $\chi$, thereby reducing the formulated heat problem to the problem with fixed boundaries (similar to how it was done in a number of publications^21,22^, when solving the problem of diffusion growth of a gas bubble).

The heat conductivity equation will take the following form:

9 $$\begin{aligned} {\bar{R}}^2\frac{\partial \Theta _l}{\partial \tau } = \ \left( -\dfrac{\alpha }{\chi ^2} + \dfrac{2}{\chi } + \beta \chi \right) \frac{\partial \Theta _l}{\partial \chi } + \frac{\partial ^{2}\Theta _l}{\partial \chi ^{2}}, \end{aligned}$$

where $\alpha = {\bar{v}}_{l R} {\bar{R}}$ and $\beta = \acute{{\bar{R}}} {\bar{R}}$ represent the functions of time; $\acute{\square } \equiv {\hbox {d}(\square )}/{\hbox {d}\uptau }$ is the function $\square$ derivative by dimensionless time $\tau$.

Initial and boundary conditions:

10 $$\begin{aligned} (\Theta _l)_{\tau =0} = 0; \quad (\Theta _l)_{\chi =1} = \Theta ^s({\bar{\rho }}_v); \quad (\Theta _l)_{\chi \rightarrow \infty } = 0. \end{aligned}$$

In the general case, the boundary-value problem (9), (10) (even without consideration of inertial effects) can be solved only numerically. However, there is a way that allows us to find a very good approximate solution. To do this, we find a steady-state solution of Eq. (9), taking into account the boundary conditions (10):

11 $$\begin{aligned} \Theta _l(\tau , \chi ) = \Theta ^s({\bar{\rho }}_v) \, \dfrac{\textit{I}(\chi , \alpha , \beta )}{\textit{I}(1, \alpha , \beta )}, \end{aligned}$$

where $$\textit{I}(\chi , \alpha , \beta ) = \displaystyle \int _{0}^{1/\chi } \exp \left\{- \alpha \zeta - \beta \zeta ^{-2}/2 \right\} \, d\zeta$$.

Numerical calculations show that the time of establishing a quasi-stationary state (in variables $\tau$ and $\chi$) is extremely short on the scale of characteristic time of the whole process. This means that solution (11) describes with good accuracy the dynamics of a temperature field forming around a bubble throughout the entire process.

Making the initial system of Eqs. (2)–(5) dimensionless and using the found temperature profile (11) in the dimensionless Eq. (5), we obtain a system of differential equations of the first order:

12 $$\begin{aligned} \begin{aligned} \acute{\alpha }&= \Pi ^s({\bar{\rho }}_v) - \dfrac{2}{{ We}}\dfrac{1}{{\bar{R}}} - \dfrac{4}{\textit{Re}} \dfrac{\alpha }{{\bar{R}}^2} - \dfrac{\alpha ^2}{2{\bar{R}}^2}; \\ \acute{{\bar{\rho }}}_v&= 3 \{[1 - {\bar{\rho }}_v]\, \beta (\alpha , {\bar{\rho }}_v) - \alpha \}/{\bar{R}}^2; \\ \acute{{\bar{R}}}&= \beta (\alpha , {\bar{\rho }}_v)/{\bar{R}}, \end{aligned} \end{aligned}$$

where function $\beta (\alpha , {\bar{\rho }}_v)$ is implicitly set by equation

13 $$\begin{aligned} (\beta - \alpha ) \textit{I}^*(\alpha , \beta ) = -\textit{Ku}^{-1} \, \Theta ^s({\bar{\rho }}_v), \end{aligned}$$

and functions $\Pi ^s({\bar{\rho }}_v)$ and $\Theta ^s({\bar{\rho }}_v)$ are defined by Eqs. (7), (8). Here $\textit{Ku}= \dfrac{{\mathscr {L}}}{c_l \Delta T_i}$ is the Kutateladze number, $\textit{We}= \dfrac{\rho _l v_0^2 R_0}{\sigma }$ is the Weber number, $\textit{Re}= \dfrac{\rho _l v_0 R_0}{\eta _l}$ is the Reynolds number, $\textit{I}^*(\alpha , \beta ) = \displaystyle \int _{0}^{1} \exp \left\{ \alpha (1 - \zeta ) + \beta (1 - \zeta ^{-2})/2 \right\} \, d\zeta$. In literature the Jacob number $\textit{Ja}= ({\bar{\rho }}_{vf} \, { Ku})^{-1} = \dfrac{\rho _l}{\rho _{vf}} \dfrac{c_l \Delta T_i}{{\mathscr {L}}}$ is often used instead of the Kutateladze number.

If vapor is considered an ideal gas, then the Mendeleev–Clapeyron equation should be used as the equation of state: $\rho _v = p_v/ (R_g T_v)$, where $R_g = {\mathscr {R}}/M$ is the reduced gas constant; ${\mathscr {R}}$ is the universal gas constant; *M* is the molar mass of vapor. In the dimensionless form it is written as follows:

14 $$\begin{aligned} {\bar{\rho }}_v = {\bar{\rho }}_{vi}\, \dfrac{1 + \varkappa _2 \Pi _v}{1 + \varkappa _1 \Theta _v}, \end{aligned}$$

where $$\varkappa _1 = \Delta T_i/T_{l i}$$; $$\varkappa _2 = \Delta p_i/p_{l i}$$; $$\rho _{vi} = p_{l i}/({\mathscr {R}} T_{l i})$$.

The Clapeyron–Clausius equation takes form: $\displaystyle \frac{\hbox {d}p^s}{\hbox {d}T_v} = \dfrac{{\mathscr {L}}}{T_v ( \rho _v^{-1} - \rho _l^{-1} )}$. At ${\bar{\rho }}_v \ll 1$, this equation can be integrated: $p^s = p_{l i} \exp \left\{ \dfrac{{\mathscr {L}}}{R_g} \left( \dfrac{1}{T^s(p_{l i})} - \dfrac{1}{T_v} \right) \right\}$. In the dimensionless from it is written as:

15 $$\begin{aligned} 1 + \varkappa _2 \Pi ^s = \exp \left\{ \varepsilon \left( (1 - \varkappa _1)^{-1} - (1 + \varkappa _1 \Theta _v)^{-1} \right) \right\} \quad {\text {or}} \quad 1 + \varkappa _1 \Theta ^s = \left\{ (1 - \varkappa _1)^{-1} - \varepsilon ^{-1} \ln ( 1 + \varkappa _2 \Pi _v ) \right\} ^{-1}, \end{aligned}$$

where $$\varepsilon = {\mathscr {L}}/(R_g T_{l i})$$.

Substituting (15) into (14), we obtain explicit dependencies ${\bar{\rho }}_v(\Pi ^s)$ and ${\bar{\rho }}_v(\Theta ^s)$, from which we can obtain implicit dependencies $\Pi ^s({\bar{\rho }}_v)$ and $\Theta ^s({\bar{\rho }}_v)$ and which should be used in Eqs. (12), (13).

Thus, the problem is reduced to solving the system of three ordinary differential equations of the form $\acute{\mathbf{y }} = \mathbf{f }(\mathbf{y })$, where $\mathbf{y } = (\alpha , {\bar{\rho }}_v, {\bar{R}})$ is the desired vector function, and function $\mathbf{f }(\mathbf{y })$ is set by the right side of Eq. (12).

We should note that integral function $\textit{I}^*(\alpha , \beta )$ has obvious asymptotic approximations, and in some cases they can be used to simplify the analysis of the resulting solution:

- at $$\alpha , \beta \ll 1$$: $$\textit{I}^*(\alpha , \beta ) \approx 1$$;
- at $$\alpha , \beta \gtrsim 1$$: $$\textit{I}^*(\alpha , \beta ) \approx \dfrac{\sqrt{\pi }}{\sqrt{2\beta + 4 \alpha - 4}}\, \exp \left\{ \left( \dfrac{\beta - \alpha + 2}{\sqrt{2\beta + 4 \alpha - 4}}\right) ^2\right\} \, \text {erfc} \left( \dfrac{\beta - \alpha + 2}{\sqrt{2\beta + 4 \alpha - 4}}\right)$$,

where $$\text {erfc}(z) = \displaystyle \int _{z}^{\infty } e^{-\zeta ^2} \, d\zeta$$.

**Figure 1:**
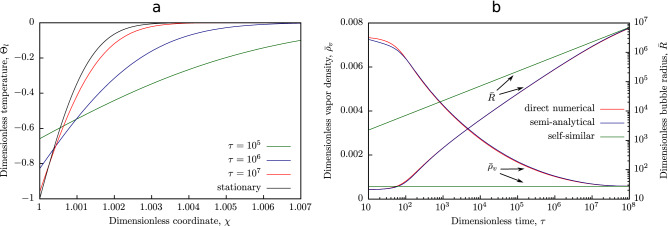
(**a**) Dependence of liquid temperature $\Theta _l$ on radial coordinate $\chi$ at various time moments $\tau$; dashed line corresponds to the stationary state achieved at large $\tau$ (thermal stage of the process). (**b**) Dependence of bubble radius ${\bar{R}}$  and vapor density ${\bar{\rho }}_v$ in the bubble on time $\tau$: solid line—numerical solution of boundary problem (2)–(6); dashed line — the semi-analytical solution (12), (13); dash-dotted line—the self-similar solution (16) describing the thermal stage of the process.

**Figure 2:**
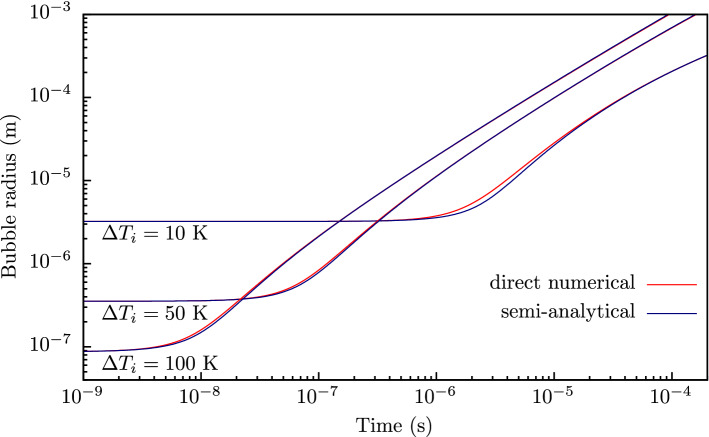
Dependence of bubble radius *R* on time *t* for different initial superheating of water: solid line—numerical solution of boundary problem (2)–(6); dashed line — the semi-analytical solution (12), (13).

Fig. 1 illustrates the the dynamics of the processes involved in bubble growth in water under the atmospheric pressure and initial superheating 100 K. It can be seen from the Fig. 1a that the temperature field with time tends asymptotically to the stationary distribution (in variables $\tau$, $\chi$), when the vapor pressure in the bubble is almost equal to the pressure of surrounding liquid.

Time dependencies of the bubble radius and vapor density in the bubble, plotted on the basis of the found semi-analytical solution (12), (13), are presented in Fig. 1b. These dependencies clearly illustrate the features of the growth mechanism of a vapor bubble in superheated liquid, combining the dynamic and thermal effects. We should note that the difference in the calculation results obtained by direct numerical simulation and on the basis of Eqs. (12), (13) turned out to be extremely small, which indicates the consistency of the found semi-analytical solution. Dependence of the bubble radius on time, constructed within the framework of the thermal energy (see the next paragraph) is also shown in Fig. 1b. The form of the plotted curves emphasizes once again that this model is essentially asymptotic and does not describe the considered process in the entire time range (as it was mentioned in Introduction).

Figure 2 shows the applicability of the found semi-analytical solution for a wide range of initial superheating. The chosen values of initial superheating $\Delta T_i = 10$ K, $\Delta T_i = 50$ K and $\Delta T_i = 100$ K approximately correspond to the values of Jacob number $\textit{Ja}$ of 30, 160 and 320 respectively. It can be seen that the results of direct numerical calculation of the boundary problem (2)–(6) differ insignificantly from the results obtained using found semi-analytical solution (12), (13) for all values of initial superheating under consideration. This means that obtained semi-anlytical solution can be used as an alternative to direct numerical calculations in solving the problems involving vapor bubble growth.

### Thermal stage: self-similar solution

According to the simple analysis of solution (12), (13) obtained above, as the bubble grows, the pressure in it decreases gradually and tends asymptotically to the pressure of the surrounding liquid ($\Pi _v \rightarrow 0$), and the vapor density in the bubble and the liquid temperature at the bubble boundary tend to the certain constant values: ${\bar{\rho }}_v \rightarrow {\bar{\rho }}_{vf}$; $(\Theta _l)_{\chi =1} \rightarrow -1$, where $\rho _{vf}$ is the vapor density at pressure $p_{l i}$ (calculated on the saturation line). It should be noted that if we do not take into account any exotic cases, then ${\bar{\rho }}_{vf} \ll 1$. At this stage of the process, called the thermal stage, which is essentially asymptotic and can be described in terms of the energy thermal model, the bubble growth is determined exclusively by heat supply to the interface, the temperature field around the bubble becomes stationary, functions $\alpha (\tau )$ and $\beta (\tau )$ become constant, and the solution to the boundary value problem becomes self-similar (the self-similar variable here is variable $\chi$, and in this case it is proportional to dimensionless complex $r/\sqrt{a_l t}$):

16 $$\begin{aligned} \Theta _l(\chi ) = -\dfrac{\textit{I}(\chi , \kappa \beta _f, \beta _f)}{\textit{I}(1, \kappa \beta _f, \beta _f)}; \quad {\bar{R}} = \sqrt{2\beta _f \tau }, \end{aligned}$$

where $\kappa = 1 - {\bar{\rho }}_{vf}$. The similar solution was obtained by Scriven ^4^.

The coefficient $\beta _f$ occurring in Eq. (16) at this stage is a function of only Jacob number and is found from the implicit equation (which follows directly from Eq. (13)):

17 $$\begin{aligned} \beta _f\, \textit{I}^*(\kappa \beta _f, \beta _f) = \textit{Ja}. \end{aligned}$$

It should be noted that in solution (16), (17), the temperature field does not depend explicitly on time; therefore, an assumption about the quasi-stationary nature of the process (in variables $\tau$, $\chi$) made above is satisfied here automatically.

In the case of low and high superheating, in the approximation of ${\bar{\rho }}_{vf} \approx 0$, which is often used in the literature, there are obvious asymptotic approximations. For $\textit{Ja}\ll 1$: $\beta _f \approx \textit{Ja}$. For $\textit{Ja}\gg 1$: $\beta _f \approx (6/\pi )(\textit{Ja}+ 4/9)^2$. The latter coincides with the well-known Plesset–Zwick solution ^1^ (if we do not take into account correction 4/9 to the Jacob number, which significantly improves this asymptotic approximation).

**Figure 3:**
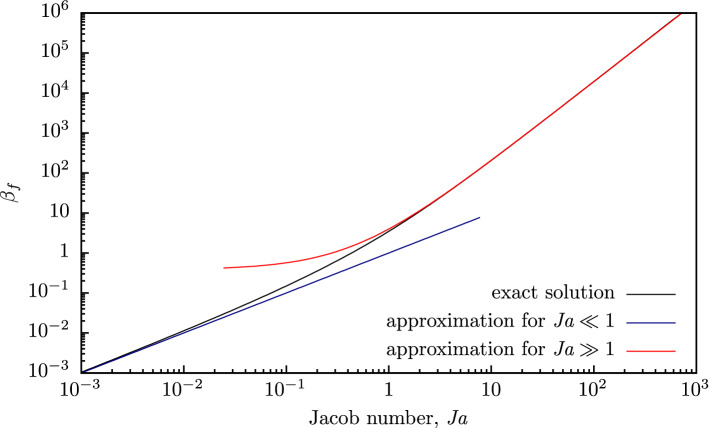
Dependence of $\beta _f$ on Jacob number $\textit{Ja}$: solid line—exact solution (17); dash-dotted line — approximate solution for large $\textit{Ja}$; dashed line—approximate solution for small $\textit{Ja}$.

Figure 3 illustrates the dependence of coefficient $\beta _f$ on Jacob number $\textit{Ja}$ calculated by formula (17) and using asymptotic approximations. Let us note that there is some discrepancy between solution (17) and asymptotic approximations at high Jacob numbers, since the latter was obtained with the assumption of ${\bar{\rho }}_{vf} \approx 0$, which is not entirely allowable for $\beta _f \gtrsim 1/{\bar{\rho }}_{vf}$.

From the above analysis, we can conclude that due to the good correspondence of direct numerical and approximation solutions, the thermal stage of the vapor bubble growth has been studied in sufficient detail in the literature.

## Conclusions

A mathematical model of the vapor bubble growth in initially uniformly superheated liquid has been formulated. This model takes into account simultaneously the dynamic and thermal effects and includes the well-known classical equations: the Rayleigh equation and the energy equation, written in relation to the studied problem with consideration of the specifics associated with the process of liquid evaporation. The following assumptions were used: the temperature and pressure of vapor in the bubble are homogeneous; vapor in the bubble is stationary and it is in a saturated state throughout the process.

It is shown that the presented problem can be reduced to solving a system of three ordinary differential equations of the first order: a new semi-analytical solution of the problem, which simplifies the analysis of the process greatly. It is shown that the solution obtained is in good agreement with direct numerical calculations in a wide range of superheating and at all stages of the process, including the transition one, which must be taken into account, especially if bubble growth in a strongly superheated liquid is considered. The dynamics of changes in the temperature field in the volume of liquid and gas density in the bubble is illustrated. The time dependence of the bubble radius is found.

It is shown that at large times the process of bubble growth is determined exclusively by the supply of heat to the interface. The temperature field around the bubble in the variables proposed in the work becomes stationary, and the solution to the thermal problem becomes self-similar. The dependence of the bubble radius on time takes the root form, and the proportionality coefficient depends only on the Jacob number (which corresponds to the well-known solutions of other authors). However, as calculations show, for certain operating parameters of the process (in particular, for high Jacob numbers), this stage is unattainable during the real bubble growth processes.

Thus, a relatively simple semi-analytical solution to the problem of the vapor bubble growth in an initially uniformly superheated liquid is found. It is constructed on the basis of the existence of a quasi-stationary state for the bubble growth process. This allows the original boundary value problem with a moving boundary to be reduced to a system of ordinary differential equations of the first order. It should be noted that this solution accounts simultaneously for both the inertial and thermal effects that control the process. It can become the better alternative to direct numerical calculations, since the use of the found analytical solution removes the need for resource-intensive numerical simulation of the heat problem.

## References

[1] Plesset MS, Zwick SA (1954). The growth of vapor bubbles in superheated liquids. J. Appl. Phys..

[2] Forster HK, Zuber N (1954). Growth of a vapor bubble in a superheated liquid. J. Appl. Phys..

[3] Birkhoff G, Margulies RS, Horning WA (1958). Spherical bubble growth. Phys. Fluids.

[4] Scriven LE (1959). On the dynamics of phase growth. Chem. Eng. Sci..

[5] Mikic BB, Rohsenow WM, Griffith P (1970). On bubble growth rates. Int. J. Heat Mass Transf..

[6] Prosperetti A, Plesset MS (1978). Vapour-bubble growth in a superheated liquid. J. Fluid Mech..

[7] Robinson AJ, Judd RL (2004). The dynamics of spherical bubble growth. Int. J. Heat Mass Transf..

[8] Yang H, Desyatov AV, Cherkasov SG, McConnell DB (2008). On the fulfillment of the energy conservation law in mathematical models of evolution of single spherical bubble. Int. J. Heat Mass Transf..

[9] Zou A, Chanana A, Agrawal A, Wayner PC, Maroo SC (2016). Steady state vapor bubble in pool boiling. Sci. Rep..

[10] Wang Q, Gu J, Li Z, Yao W (2017). Dynamic modeling of bubble growth in vapor-liquid phase change covering a wide range of superheats and pressures. Chem. Eng. Sci..

[11] Prosperetti A (2017). Vapor bubbles. Annu. Rev. Fluid Mech..

[12] Cai C, Liu H, Xi X, Jia M, Yin H (2018). Bubble growth model in uniformly superheated binary liquid mixture. Int. J. Heat Mass Transf..

[13] Arienti M, Hwang J, Pickett L, Shekhawat Y (2020). A thermally-limited bubble growth model for the relaxation time of superheated fuels. Int. J. Heat Mass Transf..

[14] Lee HS, Merte H (1996). Spherical vapor bubble growth in uniformly superheated liquids. Int. J. Heat Mass Transf..

[15] Aktershev SP (2005). Vapor bubble growth in a liquid at the superheat limit temperature. Thermophys. Aeromech..

[16] Lajoinie G (2014). Ultrafast vapourization dynamics of laser-activated polymeric microcapsules. Nat. Commun..

[17] Sadeghi R, Shadloo MS, Jamalabadi MYA, Karimipour A (2016). A three-dimensional lattice Boltzmann model for numerical investigation of bubble growth in pool boiling. Int. Commun. Heat Mass Transf..

[18] Emery TS, Raghupathi PA, Kandlikar SG (2018). Bubble growth inside an evaporating liquid droplet introduced in an immiscible superheated liquid. Int. J. Heat Mass Transf..

[19] Proussevitch AA, Sahagian DL (1998). Dynamics and energetics of bubble growth in magmas: analytical formulation and numerical modeling. J. Geophys. Res.: Solid Earth.

[20] Gor, G. Y. & Kuchma, A. E. Dynamics of gas bubble growth in a supersaturated solution with sievert’s solubility law. *J. Chem. Phys.* **131**, (2009). 10.1063/1.3176896 19624209

[21] Chernov, A. A., Kedrinsky, V. K. & Pil’nik, A. A. Kinetics of gas bubble nucleation and growth in magmatic melt at its rapid decompression. *Phys. Fluids ***26**, 116602 (2014).

[22] Chernov AA, Pil’nik AA, Davydov MN, Ermanyuk EV, Pakhomov MA (2018). Gas nucleus growth in high-viscosity liquid under strongly non-equilibrium conditions. Int. J. Heat Mass Transf..

[23] Kuchma AE, Shchekin AK, Martyukova DS, Savin AV (2018). Dynamics of ensemble of gas bubbles with account of the laplace pressure on the nucleation stage at degassing in a gas-liquid mixture. Fluid Phase Equilib..

[24] Qin Y, Wang Z, Zou L, He M (2019). Semi-numerical, semi-analytical approximations of the Rayleigh equation for gas-filled hyper-spherical bubble. Int. J. Comput. Methods.

[25] Zudin YB (2019). Non-equilibrium Evaporation and Condensation Processes.

[26] Yagov VV (1988). On the limiting law of growth of vapor bubbles in the region of very low pressures (high Jakob numbers). High Temp..

[27] Avdeev AA (2014). Laws of vapor bubble growth in the superheated liquid volume (thermal growth scheme). High Temp..

[28] Nigmatulin, R. I. *Dynamics of Multiphase Media* (Hemisphere Publ, 1990).

